# Semantic segmentation of PolSAR image data using advanced deep learning model

**DOI:** 10.1038/s41598-021-94422-y

**Published:** 2021-07-28

**Authors:** Rajat Garg, Anil Kumar, Nikunj Bansal, Manish Prateek, Shashi Kumar

**Affiliations:** 1 School of Computer Science, University of Petroleum and Energy Studies, Dehradun, Uttarakhand 248007 India; 2 Dev Bhoomi Group of Institutions, Dehradun, Uttarakhand 248007 India; 3 Photogrammetry and Remote Sensing Department, Indian Institute of Remote Sensing (IIRS), ISRO, 04 Kalidas Road, Dehradun, Uttarakhand 248001 India

**Keywords:** Engineering, Computational science, Computer science, Scientific data, Statistics, Environmental impact, Astronomical instrumentation, Information theory and computation

## Abstract

Urban area mapping is an important application of remote sensing which aims at both estimation and change in land cover under the urban area. A major challenge being faced while analyzing Synthetic Aperture Radar (SAR) based remote sensing data is that there is a lot of similarity between highly vegetated urban areas and oriented urban targets with that of actual vegetation. This similarity between some urban areas and vegetation leads to misclassification of the urban area into forest cover. The present work is a precursor study for the dual-frequency L and S-band NASA-ISRO Synthetic Aperture Radar (NISAR) mission and aims at minimizing the misclassification of such highly vegetated and oriented urban targets into vegetation class with the help of deep learning. In this study, three machine learning algorithms Random Forest (RF), K-Nearest Neighbour (KNN), and Support Vector Machine (SVM) have been implemented along with a deep learning model DeepLabv3+ for semantic segmentation of Polarimetric SAR (PolSAR) data. It is a general perception that a large dataset is required for the successful implementation of any deep learning model but in the field of SAR based remote sensing, a major issue is the unavailability of a large benchmark labeled dataset for the implementation of deep learning algorithms from scratch. In current work, it has been shown that a pre-trained deep learning model DeepLabv3+ outperforms the machine learning algorithms for land use and land cover (LULC) classification task even with a small dataset using transfer learning. The highest pixel accuracy of 87.78% and overall pixel accuracy of 85.65% have been achieved with DeepLabv3+ and Random Forest performs best among the machine learning algorithms with overall pixel accuracy of 77.91% while SVM and KNN trail with an overall accuracy of 77.01% and 76.47% respectively. The highest precision of 0.9228 is recorded for the urban class for semantic segmentation task with DeepLabv3+ while machine learning algorithms SVM and RF gave comparable results with a precision of 0.8977 and 0.8958 respectively.

## Introduction

Synthetic Aperture Radar (SAR) is a type of active sensor that generates its energy which is transmitted in the form of electromagnetic waves and then receives a part of this energy after interaction with the earth’s surface^1^. In SAR based remote sensing, wavelength plays a crucial role since it determines how the wave interacts with the earth's surface. The longer is the wavelength, the higher is the penetration depth^2^. Also, the SAR sensor collects signals in different polarizations. By analyzing these different polarization signals, one can identify the structure of the imaged surface with good accuracy based upon the dominant scattering-type – odd bounce, even bounce, and volume scattering. Target decomposition methods are used for classifying the PolSAR data into different scattering types^3^. The urban land cover mapping is becoming increasingly important since rapid urbanization has caused steady degradation of urban vegetation^4^. So, accurate classification of urban land cover is required for better urban planning and to avoid overstressing nature. While the decomposition methods are used to generate a false-color composite image based on the backscatter values, they often misclassify some urban areas as vegetation due to the diffused scattering type from oriented urban targets. This misclassification can be corrected with the help of semantic segmentation tasks using advanced machine learning algorithms. Semantic Segmentation involves assigning a class label to every pixel in the image^5,6^.

A comparative study by Gupta et al.^7^ using SAR imagery between different classification algorithms like texture-based, SAR observables based, probability density function (pdf) based, and color based is done and then all of these are fused using the RF classifier. The results of this fusion based RF classifier outperform the individual classification algorithms and the overall accuracy obtained is 88%. Another comparative study by Camargo et al.^8^ using SAR imagery for land-cover classification on Brazilian Savana Forest showed that machine learning algorithms SVM, RF, and Multi Layer Perceptron (MLP) give better results compared to Naïve Bayes and J48 with SVM clocking the highest overall accuracy. On contrary, a similar study done by Lapini et al.^9^ for the Mediterranean Forest area showed that the RF classifier achieves higher accuracy compared to the SVM classifier reason could be the imbalanced number of samples among classes.

One of the core components in the image segmentation task is feature extraction. Texture descriptor matrices measure information about spatial positioning or intensity due to which they are extensively used to extract spatial features of the SAR image^10^. Texture analysis is generally divided into three categories: transform-domain analysis, statistical analysis, and model-based analysis^11^. In the transform domain analysis, wavelet transforms are used to analyze SAR images at different orientations. Because of its geometric and stochastic properties the Discrete Fourier Transform (DFT) allows for an efficient multiresolution representation of SAR images^12^ and the Gabor transform is a variant of Fourier transform, defined as the Fourier transform multiplied by a Gaussian function^13^. The commonly used statistical analysis methods include Grey-Level Co-occurrence Matrix (GLCM) and Multilevel Local Pattern Histogram (MLPH). The GLCM computes image properties related to second-order statistics like energy, entropy, contrast, homogeneity, and correlation^14^. The MLPH outlines the size distributions of dark, bright, and homogenous patterns appearing in a moving window at various contrasts^15^. In the model-based analysis, the Markov Random Field (MRF) is widely used to analyze spatial information between neighboring pixels. To represent the spatial logic relationships, the conditional random field model is integrated with a multiscale region connection calculus model^16^. In the current work, we have used the Gabor filter, Median filter, Gaussian filter, and Canny edge detector to extract the SAR image features. These features combined with the RGB bands of the false-color composite image are used to train the machine learning models for the task of semantic segmentation.

Optical multispectral remote sensing has successfully demonstrated its potential in feature identification and land use/ land cover classification^17–20^. The major limitation of optical remote sensing is its dependency on the top surface color of an object. The portion of the electromagnetic spectrum which is used in optical multispectral remote sensing is the reflection of light in the visible range (0.4–0.7 μm) and reflected infrared (0.7–3 μm). This range for optical multispectral remote sensing is limited to the top surface color information of the objects and the short wavelength that is easily blocked by the atmospheric cloud. The active imaging microwave remote sensing is carried in the 1 mm to 100 cm wavelength range of the electromagnetic spectrum, and this range is sensitive to the structural and electrical properties of an object. Synthetic aperture Radar remote sensing has the advantages of scattering-based object characterization to identify structural and electrical properties and has several other advantages in data acquisition in the darkened environment and active imaging that can acquire data for SAR-based earth observation at night time too^21^. Polarimetric Synthetic Aperture Radar (PolSAR) is an advanced technique of SAR remote sensing in which fully polarimetric properties of the transmitted and received electromagnetic waves are used to characterize the scattering behaviour of different objects/scatterers within a SAR resolution cell^22^. SAR backscatter is a coherent sum of scattering contributed by all the scatterers within a resolution cell, and the information retrieval of different scatterers within a resolution cell is not possible with single or dual-polarised data. All the four possible polarimetric combinations of PolSAR data provide necessary and required scattering information through mathematical modelling of the interaction of electromagnetic waves with different types of objects. Several polarimetric decomposition models have been developed to provide at least three scattering elements within a resolution cell^23–25^.

**Figure 1:**
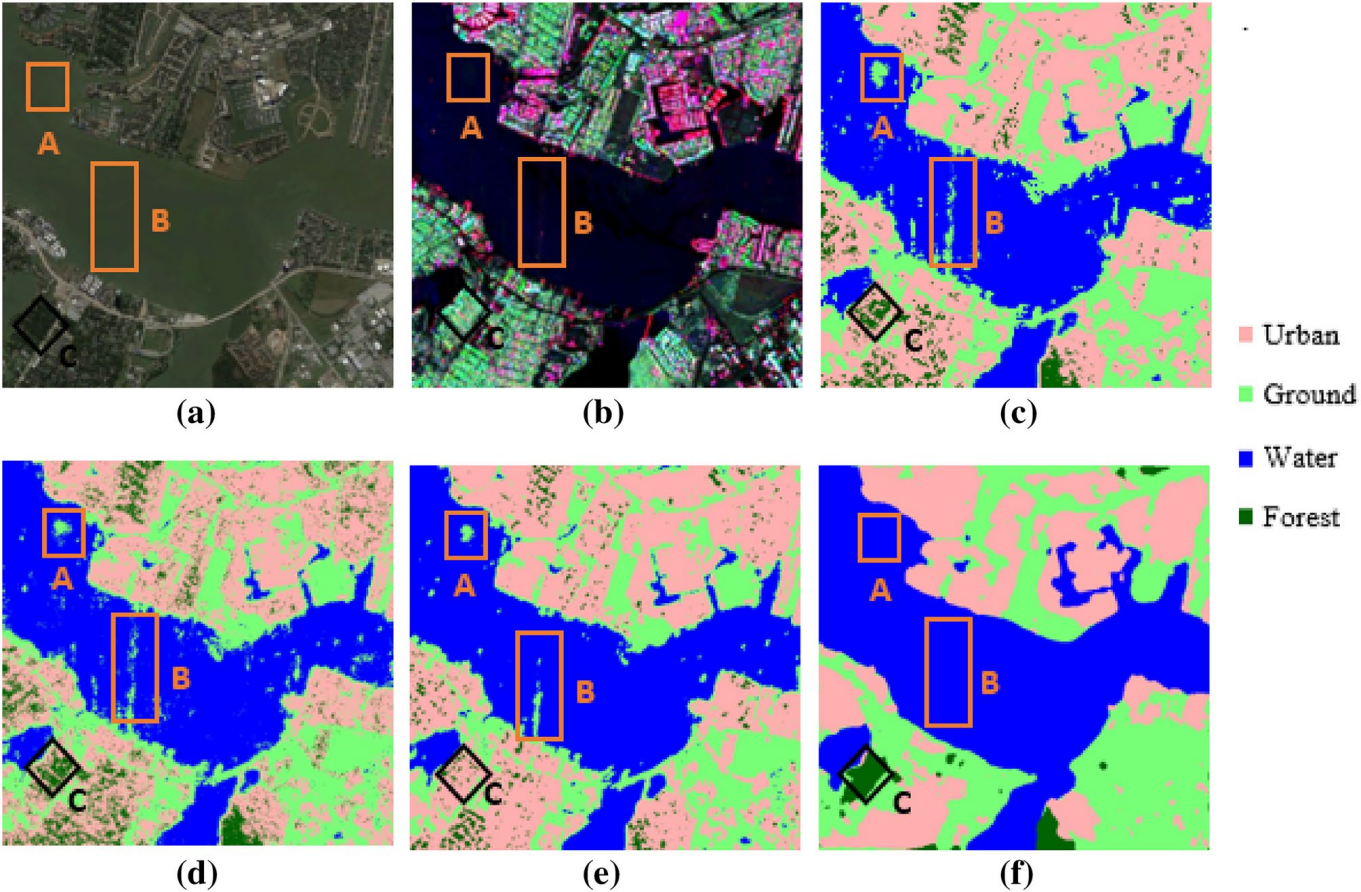
Represents the ground truth of Houston City, Texas (patch 1) and respective segmentation results of various classifiers (**a**) Google Earth image of test patch 1 (**b**) False color composite image for G4U decomposition on UAVSAR data of a part of Houston City, Texas (USA) and the corresponding image segmentation results for (**c**) RF classifier, (**d**) KNN classifier, (**e**) SVM classifier, and (**f**) Deeplab V3 respectively.

**Figure 2:**
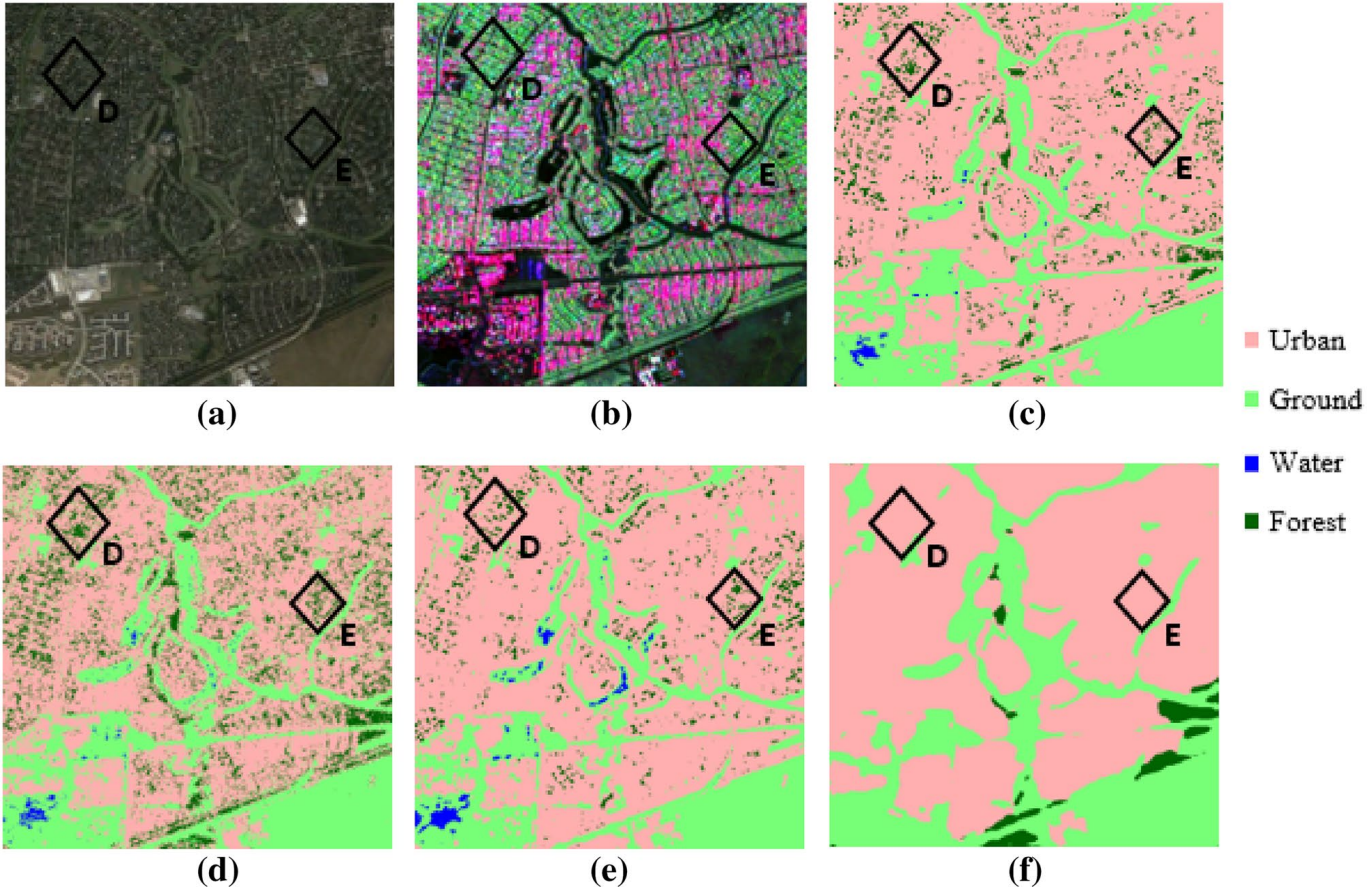
Represents the ground truth of Houston City, Texas (patch 2) and respective segmentation results of various classifiers (**a**) Google Earth image of test patch 2 (**b**) False color composite of UAVSAR image of a part of Houston City, Texas (USA) of test patch 2 and the corresponding image segmentation results for (**c**) RF classifier, (**d**) KNN classifier, (**e**) SVM classifier, and (**f**) DeepLabv3+ respectively.

The polarimetric feature is another important feature descriptor for PolSAR image segmentation. The polarimetric features are strongly dependent on scattering indexed values that are captured after a single bounce, double bounce, and volumetric scattering. The PolSAR decomposition is used to describe and classify the scattering from man-made, natural targets, and landscapes by splitting the received signal into a sum of different scattering mechanisms representing specific polarimetric signatures^23,26^. The KNN is one of the popular classifiers used for PolSAR image segmentation. The KNN is highly sensitive to the value of ‘K’ (number of neighbors). It is not efficient for a large volume of training data. A large number of polarimetric features can make it a lazy learner^27^. In Table 1, it can be observed that its training and inference time both are higher than all the other models. Overall, the KNN model is less sensitive for all the labels are shown in Fig. 1 and Fig. 2 in comparison to other models. The RF model is more robust to handle outliers and noise. The main disadvantage of the RF model is that a large number of trees and selection of other parameters can make the model too slow and may be ineffective for real-time predictions^8,28^. In Table 1, it can be observed that its inference time is more than SVM and Deeplabv3+ model. From Fig. 1 and Fig. 2, it can be observed that the RF model has good accuracies for urban and ground classification but less sensitive for water and forest. Support Vector Machine (SVM) algorithm is also a nonparametric classification model, which is insensitive to the distribution of the underlying dataset. The selection of suitable kernel, optimum kernel parameters, and the relatively complex mathematics of the SVM model restricts the effectiveness. The large training data size can also affect the performance of model^29^. From Fig. 1 and Fig. 2, it can be observed that the SVM model providing better segmentation for the water and urban class labels due to the kernel size and its sensitivity as compared to RF and KNN model. But it is less than the results of the DeepLabv3+ model.

**Table 1:** The results of performance evaluation of traditional machine learning algorithms and DeepLabv3+ algorithm.

Parameter	RF	KNN	SVM	DeepLabv3+
Pixel accuracy_Patch 1_	74.74%	78.68%	74.44%	83.51%
Pixel accuracy_Patch 2_	81.09%	74.31%	79.59%	87.78%
Overall pixel accuracy	77.92%	76.48%	77.02%	85.65%
F1 score	0.7784	0.7762	0.7646	0.8520
Precision (Urban Class)	0.8958	0.7984	0.8977	0.9228
Training time	4204 s	41,091 s	2803 s	13411 s
Inference time (per scene)	3.04 s	864.94 s	0.46 s	0.31 s
Algorithm complexity	$O\left( {V {\text{x }}NlogN} \right)$^b^	$O\left( {KND} \right)$^b^	$O\left( {N^{2} } \right)$	$O\left( N \right)$^a^

^a^It is considered only the impact of the training set of size N, however, there are other factors (number of features, dimensionality, etc.) that can also affect the algorithm’s time complexity^42,92^.

^b^In RF, the variable V = (ntree x mtry), where ‘ntree’ is the number of trees to build and ‘mtry’ represents how many variables need to sample at each node^28^. In KNN, ‘D’ is the distance between the training set observation and new observation, and ‘K’ represents the number of neighbors^27^. The variable ‘N’ represents the number of training set size used in SVM^29^ and other listed algorithms.

The Deep Neural Network (DNN) models include deep Boltzmann machines^30^, Convolutional Neural Networks^31,32^, Deep Belief Networks (DBNs)^33^, and Stacked Auto Encoders (SAEs)^34^. It has been proved that DNN models can learn higher and more abstract level of features, which can amplify the discrimination and suppress irrelevant variations in the input data^35^. Therefore, the framework of DNN improves the state-of-the-art in many computer vision tasks as well as in remote sensing applications^36,37^. Previously, Lv et al.^38^ develop a DBN for land cover mapping in urban areas using the SAR data. After that, Liu et al.^39^ designed a Wishart DBN to preliminarily classify and adopt local spatial information to adjust the classified results. Gong et al.^40^ applied a DNN to produce a change detection map directly from two images, which demonstrates an improvement in the detection performance compared with other traditional algorithms. Chen et al.^41^ proposed a Deep Convolutional Network (DCN) with sparsely connected layers for target recognition in the SAR data. This study explores some commonly used machine learning techniques along with a comparatively new semantic segmentation model DeepLabv3+^42^ to resolve the issue of misclassification up to a certain extent. To achieve the task of semantic segmentation, the DeepLabv3+ model uses an encoder-decoder network with Xception as a backbone which consists of convolution, pooling, and filtering layers. In this model, features are extracted from the backbone network Xception and atrous convolution are used in the last stage of the backbone to control the size of the feature map^40^. Here, Xception has been chosen as the backbone because it performs better compared to other architectures like RESNET-101 for the benchmark datasets like ImageNet^43^. Overall, the DeepLabv3+ model can retrieve complex textural patterns and polarimetric informative features from PolSAR image data. A detailed comparison of the models is illustrated in Table 1. One of the strong limitations of the DeepLabv3+ model is its high dependency on the amount of training data, i.e., ground truth. Although this work solved this limitation to some extent with the support of the transfer learning technique.

After the improved performance of deep learning methods in the field of computer vision, researchers from the remote sensing community have made a great effort to implement land use land cover classification using deep learning. Land use land cover maps of an area helps users and remote sensing community to understand the current landscape. It enables the monitoring of temporal dynamics of forest conversions, agricultural ecosystem, surface water bodies, biomass estimation, helps to minimize the human and economic loss from floods, tsunamis, and other natural calamities. Since a large amount of data is captured by various satellite sensors on regular basis, it creates a repository of big data. To extract the information from this big data, and classify the data as per requirements, advanced deep learning models are required that must be computationally fast and cost-efficient. The objective of this work is the semantic segmentation of PolSAR data using a deep learning technique, and this work is a part of the NASA-ISRO Synthetic Aperture Radar (NISAR) mission’s L and S-band Airborne SAR Research Announcement (RA) project on Implementation of Evolutionary Computing Algorithm for Polarimetric SAR Data Processing and Classification (TEC-07)^44^.

Conclusively, this study aims to create a new dataset for semantic segmentation of PolSAR images captured from Uninhabited Aerial Vehicle Synthetic Aperture Radar (UAVSAR) for Land Use Land Cover applications and effectively implement machine learning and advanced deep learning algorithms. This has been achieved using a small dataset with the help of transfer learning. Another aim of this study is to address the problem of oriented urban targets with the help of advanced machine learning techniques and DeepLabv3+ has addressed this issue quite well.

## Results

The results of the semantic segmentation task for patch 1 and patch 2 are presented through Figs. 1 and 2 respectively where Figs. 1a and 2a presents the Google Earth images and Figs. 1b and 2b represent the false-color composite images for the selected patches. It can be observed that the DeepLabv3+ generates very smooth and accurate land cover maps compared to traditional machine learning algorithms. In Fig. 1, areas A and B corresponds to a river that falls under the water class but all the traditional algorithms have pointed a small ground patch in these two areas while the DeepLabv3+ has predicted it as water class correctly. Further, area C contains a small forest as clear from the decomposed SAR image, but it has been very sparsely captured by RF and KNN algorithms while it is almost missed by the SVM algorithm as depicted in (Fig. 1c–e). However, the forest has been correctly captured by DeepLabv3+ which is visible in Fig. 1f. It is shown in Fig. 2, area D and area E correspond to oriented urban buildings which are misclassified as vegetation (green color) in the decomposed image. This affects the prediction of traditional machine learning algorithms as well and KNN is worst affected resulting in maximum misclassification of the oriented urban targets as vegetation. However, it is clear from Fig. 2f that the DeepLabv3+ can correctly map the oriented urban targets under the urban class with minimal misclassification.

Many performance evaluation matrices are being used to evaluate the image segmentation task like Accuracy, Kappa coefficient, F1 score, and so on. In this work, a pixel accuracy measure has been used to evaluate the performance of the semantic segmentation task. In addition to pixel accuracy, the F1 score has been reported for the resulting land cover maps. The Precision metric for the urban class has also been reported since one of the aims of this work is to address the issue of oriented urban targets which are generally misclassified as vegetation.

**Figure 3:**
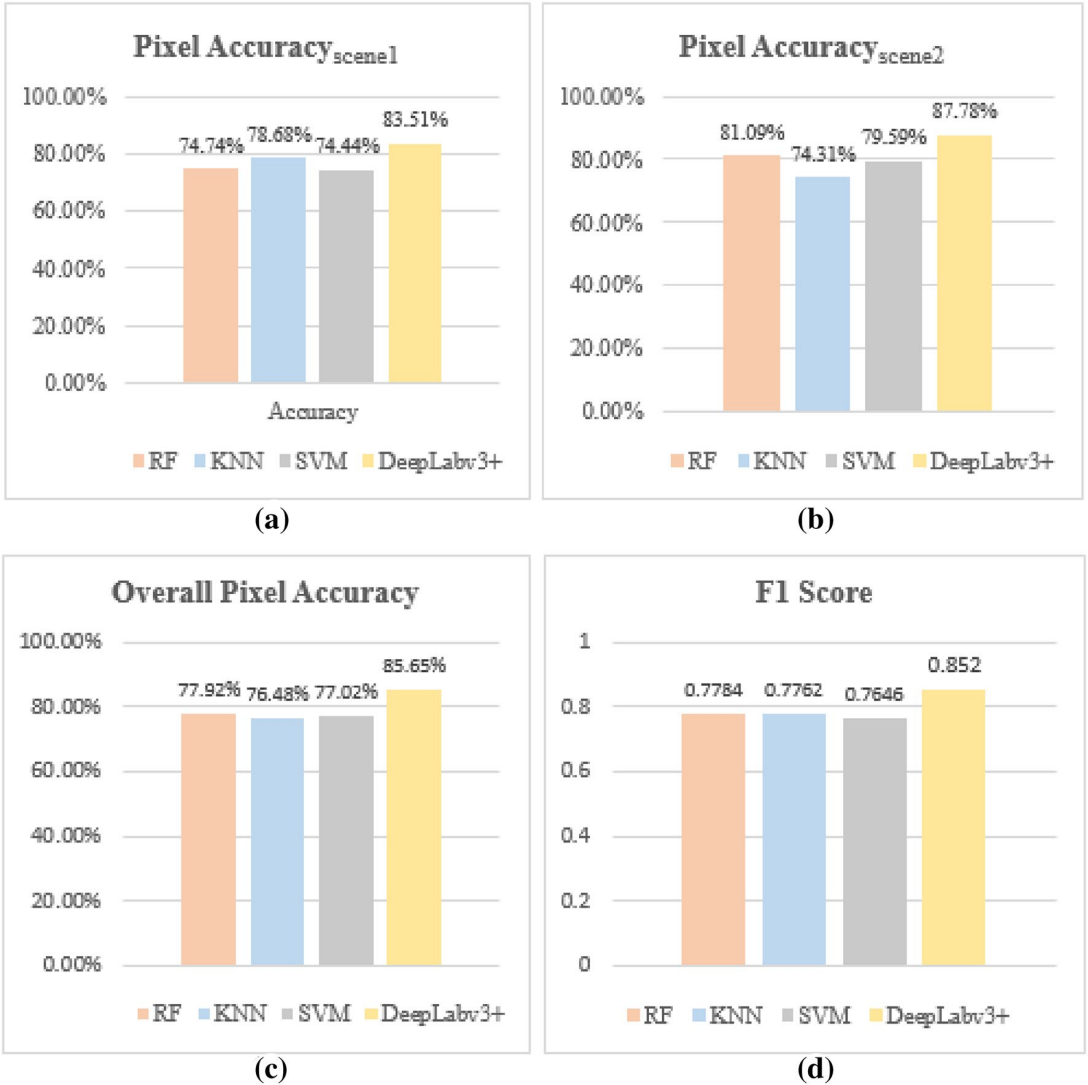
Represents the performance measures. (**a**) and (**b**) represents the pixel accuracy for scene 1 and scene 2 respectively; (**c**) represents overall pixel accuracy for both scenes/patches; (**d**) represents F1 score for both scenes/patches for RF, KNN, SVM, and DeepLabv3+ algorithms.

Figure 3 is representing the performance measures of the implemented algorithms. It is shown in Fig. 3a for the test patch 1 or scene 1, the highest pixel accuracy of 83.51% has been achieved by the DeepLabv3+ algorithm and the KNN classifier has achieved the highest pixel accuracy of 78.68% among machine learning algorithms with both Random forest and SVM classifiers recording a pixel accuracy of below 75% as reported in Table 1. For test patch 2 or scene 2 as shown in Fig. 2b, the highest pixel accuracy of 87.78% has been achieved by the DeepLabv3+ algorithm and the Random Forest classifier has recorded the highest pixel accuracy of 81.09% among machine learning algorithms with SVM and KNN classifiers trailing with a pixel accuracy of 79.59% and 74.31% respectively.

The highest overall pixel accuracy of 85.51% is recorded by the DeepLabv3+ algorithm shown in Fig. 3c while the machine learning algorithms Random forest, KNN, and SVM recorded overall pixel accuracies of 77.92%, 76.48%, and 77.02% respectively. It is observed that the performance of RF is improved in the case of patch 2 that contains more of the urban region as compared to patch 1 that has a good contribution from all the classes because it fails to map the river water accurately in patch 1. Also, the performance of the KNN algorithm drops in the case of patch 2, mainly because it fails to correctly pick up the oriented urban targets and mislabel them as vegetation. In terms of the F1 score, the DeepLabv3+ recorded the highest F1 score of 0.8520 while the machine learning algorithms RF, KNN, and SVM recorded the F1 score of 0.7784, 0.7762, and 0.7646 respectively as illustrated in Fig. 3d. To evaluate the performance of all the algorithms, specifically in the case of urban targets, we also calculated the overall precision for urban class and DeepLabv3+ recorded the highest precision of 0.9228 as shown in Table 1, while all the machine learning algorithms recorded a precision value of less than 0.90 for the urban class for the same region. This shows that the DeepLabv3+ model can correctly classify the oriented urban targets which are misclassified by the decomposition algorithms.

### Challenges and limitations of SAR data

A radar instrument, especially SAR, has several advantages over the optical multispectral sensor. Since the SAR sensors do not depend on Sun's illumination for Earth observation, imaging could be done day and night. The longer wavelength is an added advantage that could provide information for land/ocean features in the cloudy environment also. Apart from complements, SAR has various challenges and limitations. The main challenge in SAR data processing for land use/land cover classification and other thematic applications is to ensure distortion-free backscatter image^45^. Mainly three types of distortions affect the SAR data quality: radiometric, geometric, and polarimetric distortions. Appropriate calibration approaches are implemented to minimize the effect of distortions in the SAR imagery^46^. Radiometric calibration is performed with internal and external calibrators to find the actual return of the radar signals from the objects present on the earth's surface^46–48^. The backscatter image represents the radar cross-section obtained after scattering from all the objects within the resolution cell^49–51^. The concerned space agencies generally provide the radiometric calibration formula to ensure the correct radar return from the different object classes in the SAR data because it is not possible for all the researchers and users to evaluate the radiometric properties with the help of active and passive calibrators. Before implementing any classification approach or modelling for thematic applications, the radiometric calibration is performed to the Single Look Complex (SLC) or intensity/amplitude data^52,53^. Geometric distortions in spaceborne SAR occur mainly due to the undulating topography of the imaged surface^54^. Due to side-looking imaging characteristics of a SAR system, there is the possibility to occur layover, shadow, and foreshortening in SAR image during data acquisition of any hilly or undulating terrain. The geometric distortions mainly affect the SAR image in the range ^51^direction. The undulating terrain results in geometric distortions and affects the radiometric quality of the SAR data. The precise radiometric calibration of the SAR data requires the angle of incidence information of the incident electromagnetic waves transmitted by the SAR system on the terrain surface. Generally, the angle of incidence information is provided with SAR metadata, but it will be effective only if the topography of the surface is flat. If the terrain is undulating, then the actual angle of incidence will be different from the angle provided with SAR metadata^55,56^. To find the actual incidence angle for radiometric calibration and to minimize the geometric distortions from the SAR data, high-resolution digital elevation models are required^54,57^. The side-looking property of the SAR sensor not only gives geometric distortions in the undulating terrain but this also creates slant-range ambiguity^50^. Due to slant-range ambiguity, the actual shape and size of the ground targets could not be represented in the SAR imagery. The slant to ground range conversion of the data helps in providing actual ground information to the features imaged by the SAR system. The precise terrain correction algorithms are implemented with external high-resolution DEMs for the minimization of radiometric and geometric distortions from the SAR image^58,59^. The use of external DEM also helps in creating a simulation-based layover-shadow mask for the orthorectified SAR data of the undulating terrain^60,61^. The layover-shadow mask provides detailed information for the undulating terrain’s portion affected by the geometric distortions due to layover and shadow effect. The radiometric normalisation and Orthorectification process successfully minimize the radiometric and geometric distortions from the SAR data^54^. To retrieve all the scattering information contributed by different objects in a small area, fully polarimetric quad-pol data are used because retrieval of all the scattering elements within a resolution cell is mathematically and practically rigorous with single or dual polarised SAR data^62,63^. The remote sensing community has widely used the fully polarimetric quad-pol SAR data to implement model-based decomposition approaches in the retrieval of scattering parameters^64–66^. The accuracy in polarimetric SAR model-based scattering retrieval is affected by the polarimetric distortion of the SAR data^67,68^. The polarimetric distortions are cross talk, channel imbalance, and Faraday rotation^69,70^. Out of these three PolSAR distortions, Faraday rotation is highly effective in low-frequency spaceborne SAR data and this distortion could be removed by implementing appropriate approach^71^. Several algorithms have been developed to minimize the effect of cross talk and channel imbalance from the PolSAR data^72^. After minimising radiometric geometric and polarimetric distortions the scattering retrieved from the mathematical modelling and decomposition of the SAR data shows appropriate scattering for different objects^46,73,74^. The smooth surfaces like barren land and water body could be characterised as surface scatterers with a difference in the surface scattering power^24^. The forest vegetation shows a dominance of volume scattering in the PolSAR decomposition and the urban settlements show a very high amount of double-scattering power^26,75^. Since, distortion-free polarimetric SAR data helps in identifying the scattering of different features of the imaged area, hence the implementation of classification approaches on these data gives a fruitful result with reliable accuracy. This work implemented the land use land cover classification on fully polarimetric quad-pol UAVSAR data after implementing G4U decomposition that targeted the vegetation covers as a forest class and the urban class is representing by the combination of dense and sparse building areas that includes the rural areas, industrial plants and inner-city areas with high rise buildings. The achieved accuracy of urban class using DeepLabv3+ model is 92.28% that is a major significance of this work.

## Discussion

The present study provides an extensive analysis of popular machine learning classifiers Random Forest, K-Nearest Neighbor, and Support Vector Machine and deep learning model DeepLabv3+ for semantic segmentation of Uninhabited Aerial Vehicle Synthetic Aperture Radar (UAVSAR) data captured on a part of Houston City, Texas (USA). This fully polarimetric data with a high resolution of 2 m provides concrete information for the training of machine learning models. Another advantage of using the UAVSAR dataset is that pre-processing steps like calibration and multi-looking are not required to be performed since the data available on the NASA-JPL platform is already radiometrically calibrated and multi-looked. As mentioned earlier that the major hindrance in the large-scale accurate land cover mapping is the unavailability of a common benchmark dataset in a sufficient amount that could be used for training the deep learning model. Even if the data is available, the ground truth is unavailable most of the time and it is not possible to validate the results obtained without the availability of proper ground truth learning to the limitation of its usage. Also, labeling a large dataset for training a deep learning model from scratch is a very time-consuming and cumbersome task that can take up to hundreds of man-hours. This issue could be overcome with help of the transfer learning method by using a small dataset as shown in this work. The results obtained by the transfer learning method using a pre-trained DeepLabv3+ model are better than those obtained from the popular traditional machine learning algorithms.

Apart from the smooth overall image segmentation results, the deep learning model is also able to correctly classify the oriented-urban targets while the traditional machine learning algorithms failed to pick them up and misclassified them into vegetation class. This is because deep neural networks can fetch textural features better as compared to traditional algorithms. Upon close observation of the PolSAR data and the false color-composite images, in particular, it can be observed that although the color of oriented urban targets is similar to that of vegetation/forest region, they are texturally different from vegetated areas which are more smooth in terms of image, unlike urban features which have patterns due to the streets running between the urban blocks. The deep learning encoder based on Xception architecture can highlight this dissimilarity much better compared to traditional manual feature extraction methods resulting in the better performance of the deep learning model compared to traditional machine learning algorithms. The transfer learning method has a drawback of negative transfer because of the difference in the datasets used while training the model and the dataset used while transfer learning. It works best only when the two datasets are similar enough. Since the pre-trained model used in this study is trained on an ImageNet dataset that consists of real-life camera images, there could be some negative transfer due to the dissimilarity of the ImageNet dataset from the PolSAR data. But currently, there is no measure to determine the same. In the future, with the availability of a large amount of open-source PolSAR data, the training dataset can be increased to train a deep learning model from scratch and possibly achieve even higher accurate results.

## Conclusions

Land Use Land Cover mapping is important to address the ever-increasing environmental concerns like climate change, soil degradation, deforestation, and water pollution. But this need for highly accurate land cover maps is hindered by the unavailability of a common benchmark labeled dataset used to train the deep learning models. The present work demonstrated and proved that the transfer learning associated with deep learning models can be effectively used to generate accurate land cover maps and their classification even with a small training dataset. The transfer learning algorithms not only address the issue of unavailability of a large amount of labeled dataset in the field of SAR based remote sensing but also takes lesser computational time and resources compared to training a deep learning model from scratch. In both the targeted test patches, DeepLabv3+ clocked the highest pixel accuracy of 83.51% and 87.78% while among the machine learning algorithms RF classifier achieved the highest overall pixel accuracy of 77.92% for the segmentation task. It has also been validated through these experiments that the KNN classifier is rightly known as the lazy learner since it has taken a maximum training time of around 41,091 s for the overall training dataset and inference time per scene of around 864.94 s. One of the focused areas of this work was to reduce the misclassification of oriented urban targets as vegetation. This could be successfully achieved by using DeepLabv3+, an advanced deep learning model. It can be noted that the current state-of-the-art DeepLabv3+ model works better than the traditional machine learning methods even with small datasets contrary to the popular belief that large datasets are required for deep learning models to achieve better results as compared to traditional machine learning algorithms. In the future, this work can be extended to training and validating the DeepLabv3+ model from scratch subject to the availability of a common benchmark dataset in a sufficient amount.

## Methods

### Data

**Figure 4:**
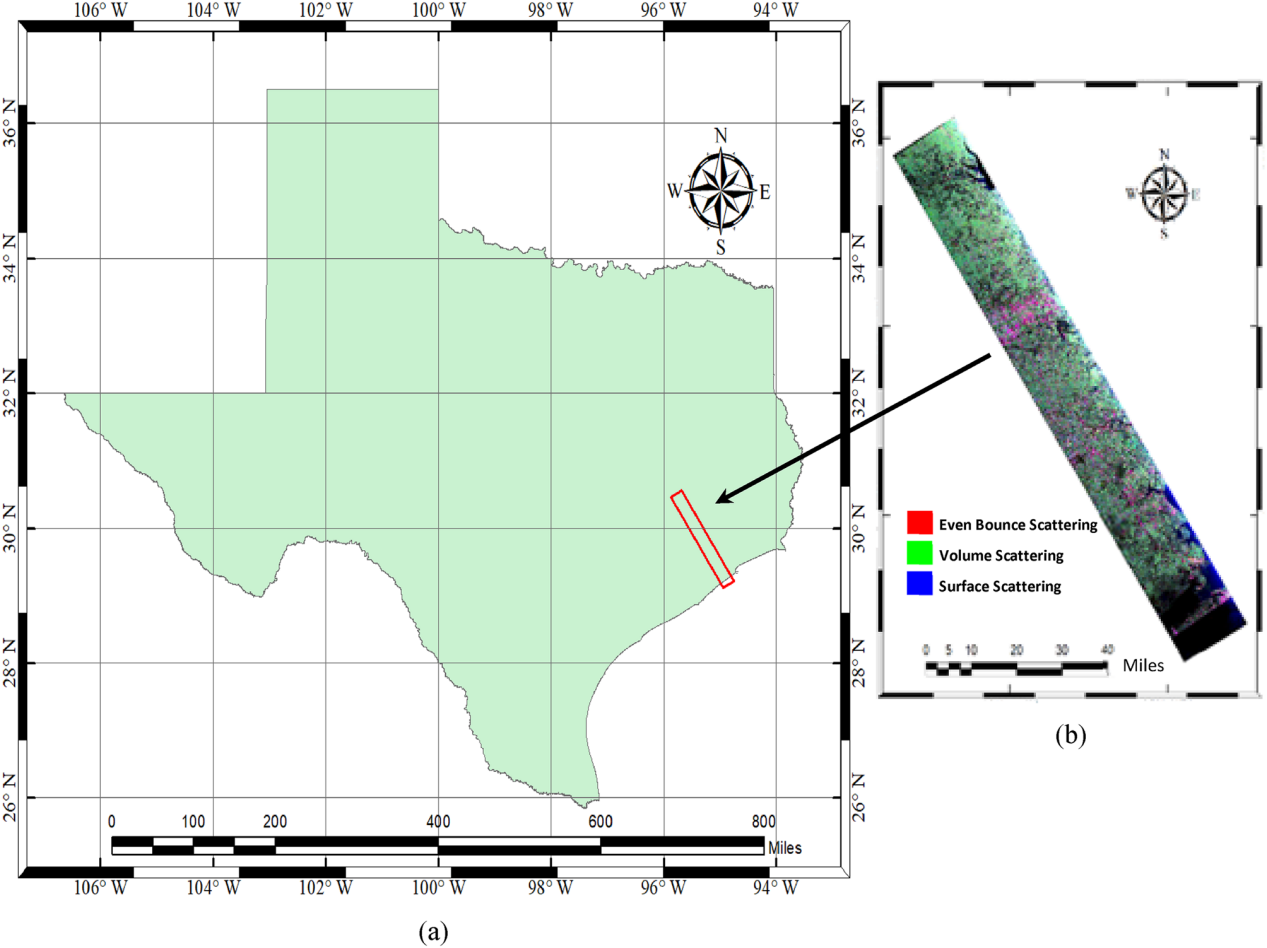
Represents the location of the study area compiled using ArcGIS v10.6 (URL: https://desktop.arcgis.com/en/arcmap/) (**a**) the boundary of Texas state (obtained from URL: https://gis-txdot.opendata.arcgis.com/) (**b**) the G4U decomposition-based false color composite image of UAVSAR data for part of Houston city, Texas (USA) generated using an open source remote sensing tool PolSARPro v6.0 (open access at URL: https://step.esa.int/main/download/polsarpro-v6-0-biomass-edition-toolbox-download/).

The data used in this research is taken from the open access platform of NASA-JPL^76^. It’s fully polarimetric L-band data from airborne sensor UAVSAR which has a resolution of 2 m. The target area of Houston City shown in Fig. 4 has been chosen as it provides a variety of land cover features. Houston is a large metropolis city in the state of Texas, USA. Most of its area consists of nearly level, clayey and loamy, prairie soils. The L-band Uninhabited Aerial Vehicle Synthetic Aperture Radar data was used to test the potential of machine learning algorithms for semantic segmentation tasks. A part of the Houston metropolis was imaged by the UAVSAR fully polarimetric quad-pol SAR data. Figure 4b shows a false-color composite image of G4U decomposition-based output for the study area. The green color highlights the features responsible for volume scattering. Double-bounce or even-bounce scatters which contribute toward high backscatter are shown in red color and the surface scattering or odd-bounce scattering is represented in blue color. The details about the UAVSAR instrument and the data used are mentioned in Table 2.

**Table 2:** Details of UAVSAR data and instrument^93^.

SAR system parameters	Details
Platform	Airborne
Flight	Uninhabited Aerial Vehicle Synthetic Aperture Radar (UAVSAR)
Date of acquisition	2 Sep 2017
Acquisition mode	PolSAR
Frequency	1.257 GHz
Wavelength	23.84035 cm
Bandwidth	80 MHz
Pulse duration	40 µs
Transmit power	3.1 kW
Look angle range	25°–65°
Data type	Ground Range Projected (equiangular) and Multi-looked Data (GRD)
Polarisation	Fully polarimetric quad-pol (HH + HV + VH + VV)
Swath width	16 km
Range resolution (m)	4.99 m (MLC product)
Azimuth resolution (m)	7.2 m (MLC product)

### Methodology

The UAVSAR Quad-Pol Ortho-Rectifying data is provided as the covariance matrix (C3) which is converted to a coherency matrix (T3). Generally, the SAR dataset is influenced by speckle noise which creates problems in object detection and classification. To minimize speckle from the PolSAR data various specialized filters are used which are mainly developed to minimize the speckle effect from polarimetric SAR data. Several filters have been developed for speckle filtering of PolSAR data where the Lee filter is one of the most famous and effective speckle filters which is used in this work for polarimetric speckle filtering of UAVSAR data. After speckle-filtering, PolSAR decomposition is applied to generate a false-color composite image which is used to create a training dataset for our ML models after breaking it into smaller patches. One of the first widely accepted model-based decompositions was proposed by Freeman and Durden^63^. This model decomposed the SAR backscatter among three different scattering types namely odd-bounce scattering, double-bounce scattering, and volume scattering. Later, an improved model with an additional scattering type, Helix scattering was proposed by Yamaguchi^77^. Based on Yamaguchi decomposition, Singh et al.^78^ proposed a general 4-component decomposition method with a unitary transformation that makes use of full polarimetric information in decomposition. In previous models, the T13 component of the Coherency matrix was not being used but this method allowed the usage of the same. It also included an extended volume scattering model which discriminates volume scattering between dihedral and dipole scattering structures caused by the cross-polarized HV component. This lead to an enhanced double-bounce scattering from urban targets compared to the existing model-based decompositions. This is the reason why the G4U decomposition method is used in the present work.

Four component decomposition is expressed in Eq. (1)

1 $$< [{\text{T}}] > = {\text{P}}_{{\text{s}}} [{\text{T}}]_{{\text{s}}} + {\text{P}}_{{\text{d}}} [{\text{T}}]_{{\text{d}}} + {\text{P}}_{{\text{v}}} [{\text{T}}]_{{\text{v}}} + {\text{P}}_{{\text{c}}} [{\text{T}}]_{{{\text{helix}}}}$$

where P_s_, P_d_, P_v_, and P_c_ are scattering powers to be determined. [T]_s_, [T]_d_, [T]_v_, and [T]_helix_ are expansion matrices corresponding to surface, double-bounce, and helix scattering respectively.

**Figure 5:**
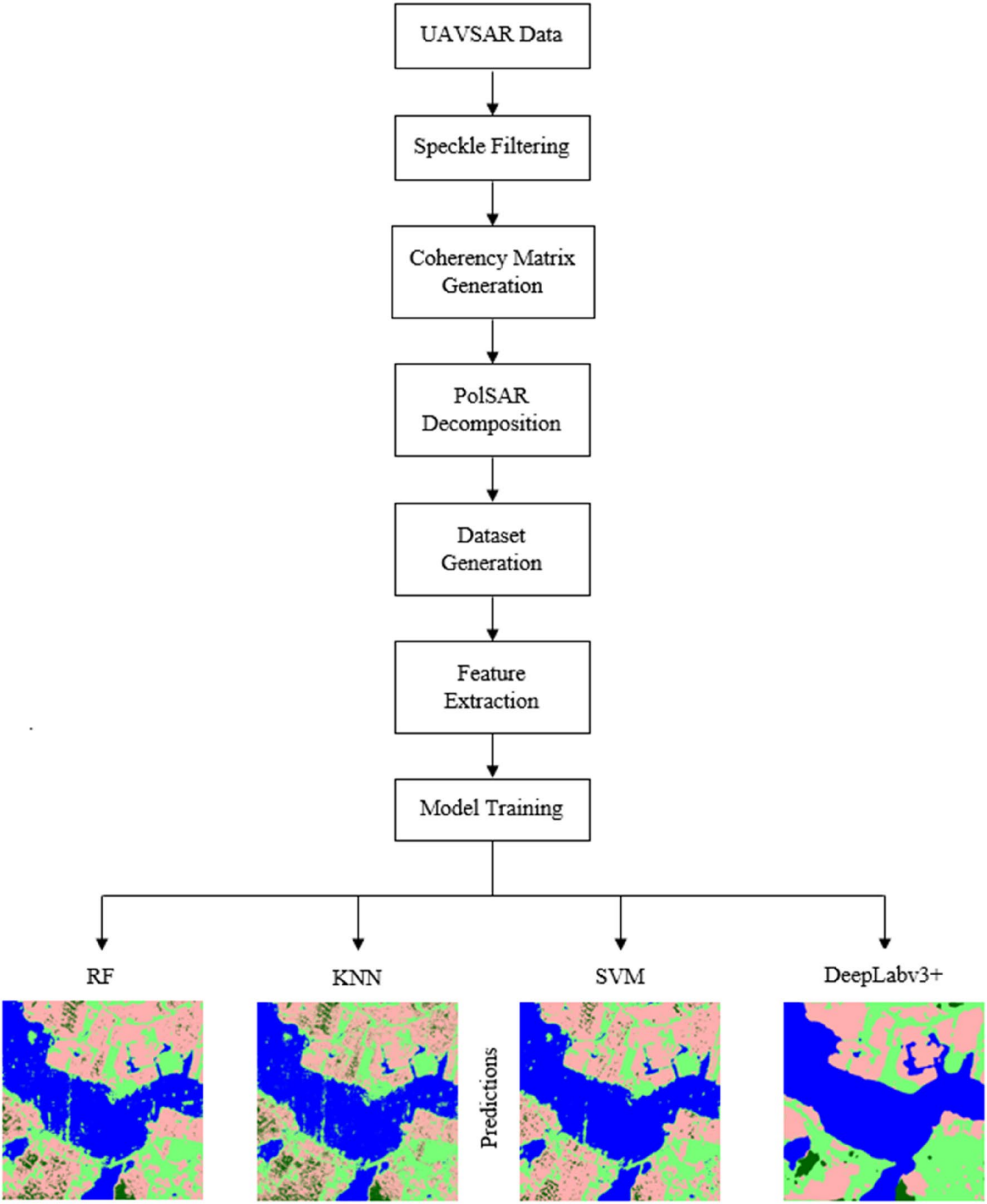
Represents the work flow of the methodology adopted for image segmentation using traditional machine learning algorithms and DeepLabv3+ model.

**Figure 6:**
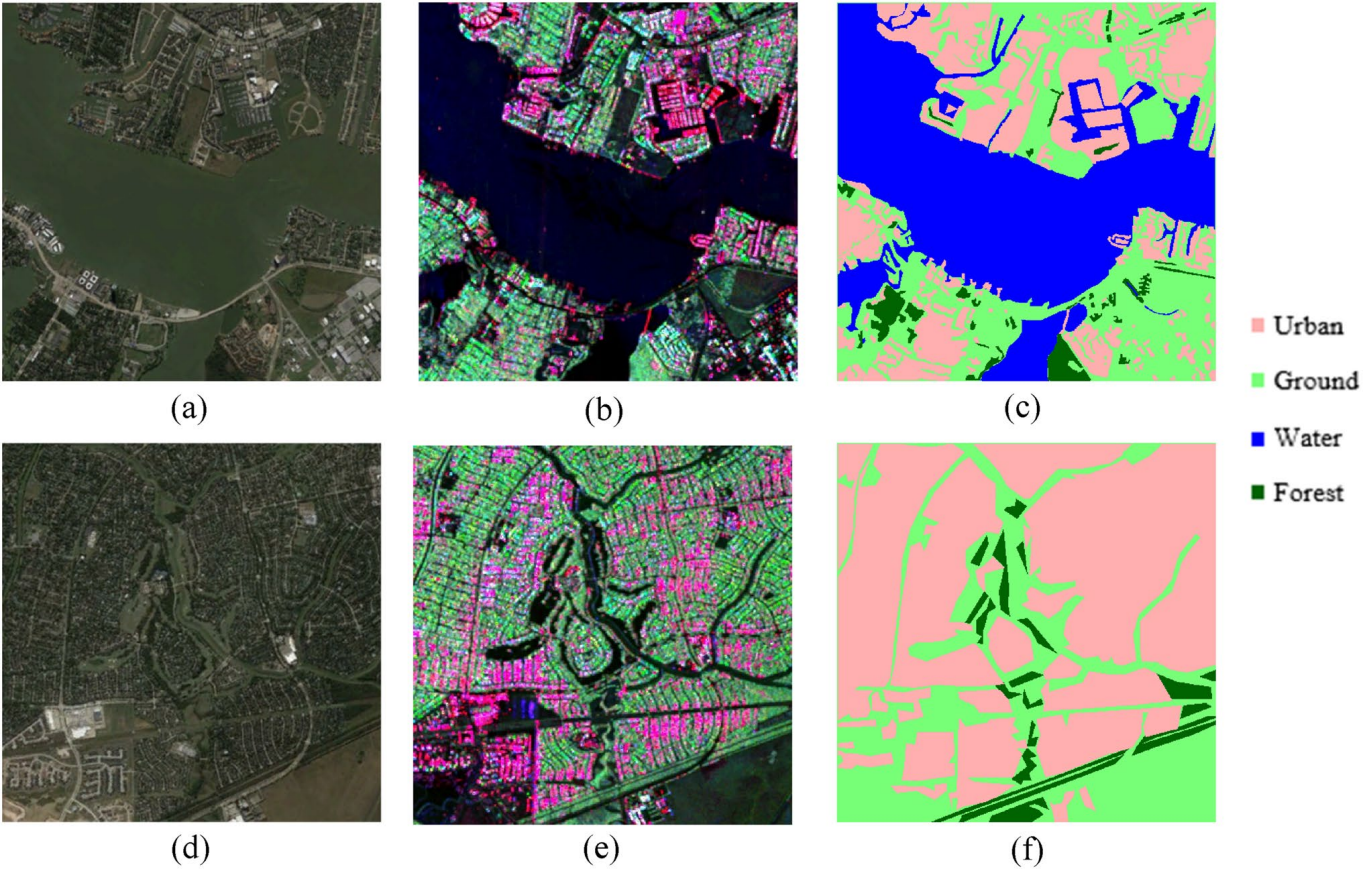
Represents the ground truth image, false-color composite image, and labeled image of the patches of Houston City, Texas(USA) (**a**) Google Earth image, (**b**) the false-color composite image, and (**c**) Ground truth label for Patch1, (**d**) Google Earth image, (**e**) the false-color composite image of G4U decomposition, and (**f**) ground truth label for Patch2.

This false-color composite image is sub-divided into smaller chunks of 500 * 500 pixels and 20 patches are labeled using the Gimp tool to create a dataset for training and testing the machine learning algorithms and the DeepLabv3+ model. From these 20 patches, 18 patches were used for training while 2 patches were kept for testing and validation. After generating the required dataset, manual feature extraction is carried out for the implementation of machine learning models by using Gabor filter, Gaussian filter, Canny edge detector, and Median filter while in the case of DeepLabv3+ feature extraction is automatically carried out by the Xception based encoder. The complete workflow adopted for the task of semantic segmentation is presented in Fig. 5 and the google image, decomposed false-color composite, and the corresponding annotated label for the test patches is shown in Fig. 6. The details about the training and working of different algorithms are discussed ahead separately.

The Gabor filter is represented by Eq. (2) which was originally given by Gabor^79^.

2 $$g\left( {x,y,T,\emptyset } \right) = h_{x} \left( {x;T,\emptyset } \right) \cdot \,h_{y} \left( {y;\emptyset } \right) = \left\{ {exp\left| {\left( {\frac{{x_{\emptyset }^{2} }}{{2\sigma_{x}^{2} }}} \right)} \right|\cos \left| {\left( {\frac{{2\pi x_{\emptyset } }}{T}} \right)} \right| \cdot \left\{ {exp\left| {\left( { - \frac{{y_{\emptyset }^{2} }}{{2\sigma_{y}^{2} }}} \right)} \right|} \right\}} \right\}$$

where $h_{x}$—Bandpass filter, $h_{y}$—Gaussian filter.

The Gaussian filter^80^ is represented by Eq. (3)

3 $$G\left( {x,y} \right) = \frac{1}{{\sqrt {2\pi \sigma } }}exp\left( { - \left( {x^{2} + y^{2} } \right)/2\sigma^{2} } \right)$$

where $\sigma^{2}$ is the variance of Gaussian filter, and $l( - l \le x,y \le l)$ is the size of the filter kernel.

In the implementation of Canny edge detector^81,82^, firstly the image undergoes Gaussian filtering and then Gradient magnitude and direction is calculated using below Eq. (4)

4 $$G\left( {i,j} \right) = \sqrt {G_{X}^{2} \left( {i,j} \right) + G_{Y}^{2} \left( {i,j} \right)} ,\quad \theta \left( {i,j} \right) = arctan\frac{{G_{x} \left( {x,y} \right)}}{{G_{y} \left( {x,y} \right)}}$$

where $G_{x} \left( {i,j} \right)$ and $G_{y} \left( {i,j} \right)$ are the partial-derivative at point $\left( {i,j} \right)$ in x-direction and y-direction respectively.

### Machine learning methods

Machine Learning is a process of enabling a computer to take independent, intelligent decisions with the help of supervised or unsupervised learning. The commonly used machine learning algorithms for classification tasks are Random Forest, K Nearest Neighbour, and Support Vector Machine.

Random forest classifier was first proposed by Breiman^28^ in 2001. It is based on Bagging predictors^83^ proposed by Breiman himself in 1996. In random forests, all the trees work independently of each other, and thus, the algorithm can be run in parallel. Random Forest classifier is very robust towards outliers and noise but it requires a large amount of training dataset. Assuming a dataset {r_1_, r_2_,…, r_n_} $\in$ R^L×n^, where L is the number of features and n is the number of samples. r_*i*_ represents the position of sample *i* in the space R^L×n^, while *z* donates the relationship between r_i_ and r_j_, where z $\in$ Z = {1,-1}. If r_i_ and r_j_ belong to the same class, then *z* = 1, and if r_i_ and r_j_ belong to different classes, then *z* = −1. For the *m*th tree, the ensemble is f_p_(r) = f(r,θ_m_), and a tree f(r,θ_m_) grows along with the training set and the random vector θ_m_, which captures the various stochastic elements of the tree. {θ_m_} are independent and identically distributed^84^.

The estimated probability for predicting class *z* for a sample is defined in Eq. (5),

5 $$P\left( {z|{\text{r}}} \right) = \frac{1}{M}\mathop \sum \limits_{m = 0}^{M} {\text{P}}_{m} (z|{\text{r}})$$

where ${\text{P}}_{m} (z|{\text{r}})$ is the estimated density of the class labels of the mth tree and M is the number of trees in the forest.

The decision function of the forest is defined in Eq. (6)

6 $${\text{C}}({\text{r}}) = {\text{arg}}\mathop {\max }\limits_{{j \in {\text{Z}}}} {\text{P}}({\text{j}}|{\text{r}})$$

The margin function for a random forest is defined in Eq. (7)

7 $${\text{ml}}\left( {{\text{r}},{\text{z}}} \right) = {\text{P}}(z|{\text{r}}) - \mathop {\max }\limits_{{j \in {\text{Z}}}} {\text{P}}_{m} (j|{\text{r}})$$

where j ≠ *z* and if ml(r,z) > 0, we can obtain a correct result.

K-Nearest Neighbor has proved to be a powerful nonparametric classifier^27^. As per the Nearest Neighbor rule, if a set of *n* pairs (*x*_1_,*θ*_1_),…., (*x*_*n*_, *θ*_*n*_) is considered, where the *x*_*i*_’s take values in a metric space *X* upon which is defined a metric *d*, and the *θ*_*i*_’s take values in the set {1, 2,…., *M*}. Every value of the *θ* set is considered as the index of the category to which *i*th individual belongs, and every corresponding *x*_*i*_ is the outcome of the set of measurements made upon that individual. Given a new pair (*x, θ*), where *x* is the measurable quantity and *θ* needs to be estimated with the help of information available from the set of correctly classified points. Then *x*’ $\in$ {*x*_*1*_, *x*_*2*_,…., *x*_*n*_} is called the nearest neighbor to *x* if it satisfies the Eq. (8),

8 $$\min d\left( {x_{i} , \, x} \right) = d(x_{n}^{^{\prime}} ,x)\quad i = 1,2, \ldots ,{\text{n}}.$$

The Nearest Neighbor rule predicts that x belongs to the category *θ*_*n*_′ of its nearest neighbor *x*_*n*_′. A mistake occurs if *θ*_*n*_′* ≠ θ*. The Nearest Neighbor rule utilizes only the classification of the nearest neighbor. The n-1 remaining classification *θ*_*i*_ are ignored.

Support Vector Machine classifier acts as a parametric classifier when used as a linear SVM while advanced SVMs used to classify complex non-linear data are non-parametric. The complex dataset can be projected as linear in a high-dimensional feature space using kernels like radial basis function (RBF), but the computation is not carried out in that high dimensional feature space. By using kernels, all necessary computations are performed directly in the input space^29^. There are three types of kernels mainly used with non-parametric SVMs – polynomial kernel, RBF kernel, and sigmoid kernel as represented by Eqs. (9), (10), and (11).

Polynomial Kernel (*k*),

9 $${\text{k}}\,{\text{(x}},{\text{y)}} = ({\text{x}} \cdot \,{\text{y}})^{{\text{d}}}$$

corresponding to a map Φ in the space spanning all products of exactly d dimensions of R^N^.

RBF Kernel (*k*),

10 $$k\left( {{\text{x}},{\text{y}}} \right) = \exp \left( { - \left| {\left| {{\text{x}} - {\text{y}}} \right|} \right|^{2} /\left( {2\upsigma ^{2} } \right)} \right)$$

where 1/2σ^2^ = γ and γ is the free parameter.

Sigmoid Kernel (*k*),

11 $$k\left( {{\text{x}},{\text{y}}} \right) = \tanh \left( {\upkappa \left( {{\text{x}} \cdot \,{\text{y}}} \right) +\uptheta } \right)$$

where κ is the gain and θ is the offset value.

### Deep learning methods

In Deep Learning, there are different approaches to implement Semantic Segmentation. The most commonly used is based on Fully Convolutional Networks (FCN). Unlike classical CNN, which has fixed fully connected networks, FCN has only convolution and pooling layers that allow it to work with arbitrarily sized inputs^85^.

A general Semantic Segmentation architecture consists of an encoder-decoder network. An encoder network can be a classification network like VGG/RESNET/Xception. A decoder network is used to project the low-resolution result of the encoder network to a high-resolution pixel space by upsampling. An encoder-decoder network has been successfully used for the semantic segmentation task^86–88^.

## DeepLabv3+

**Figure 7:**
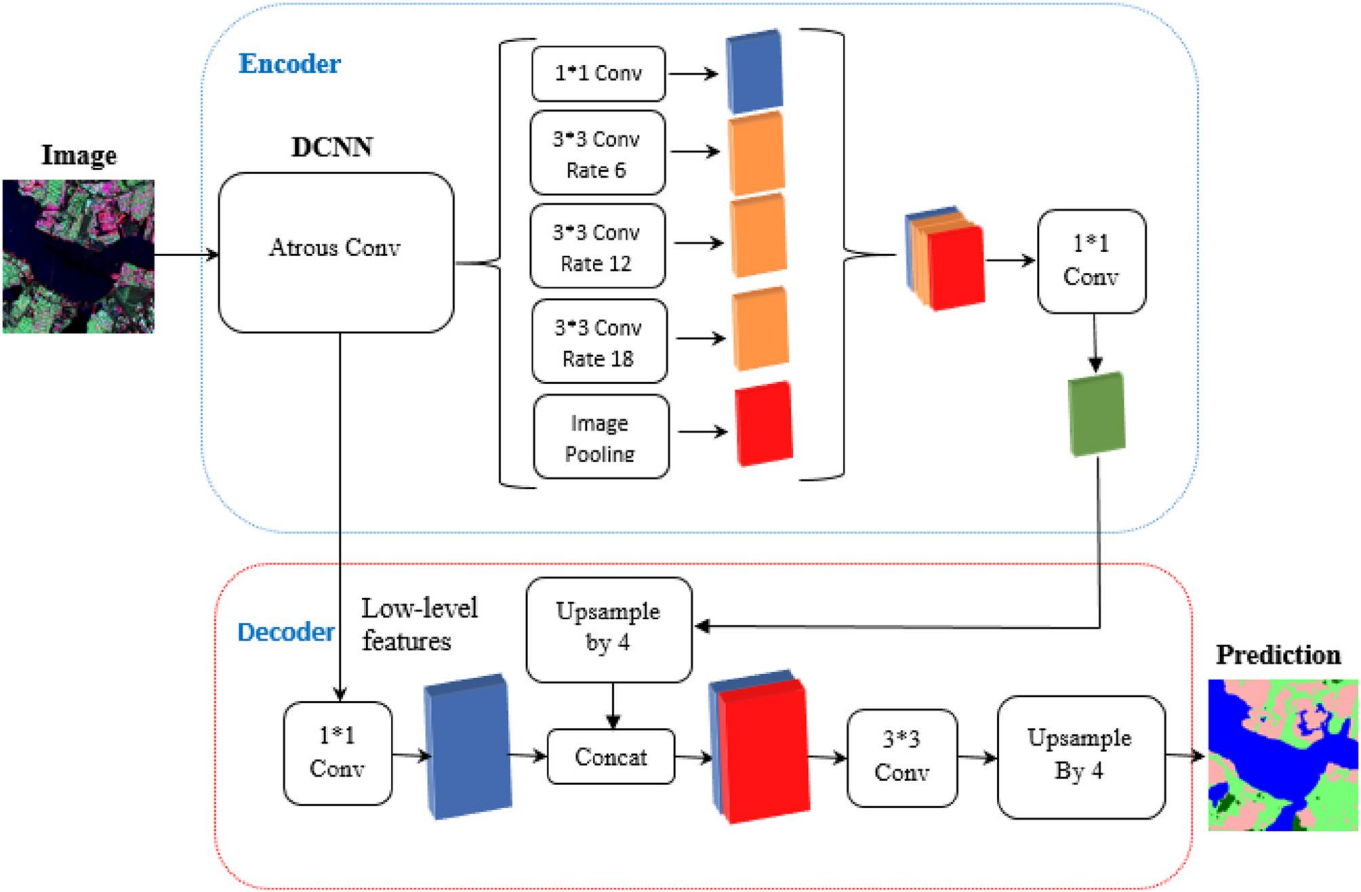
Encoder-Decoder based structure of DeepLabv3+^42^.

**Figure 8:**
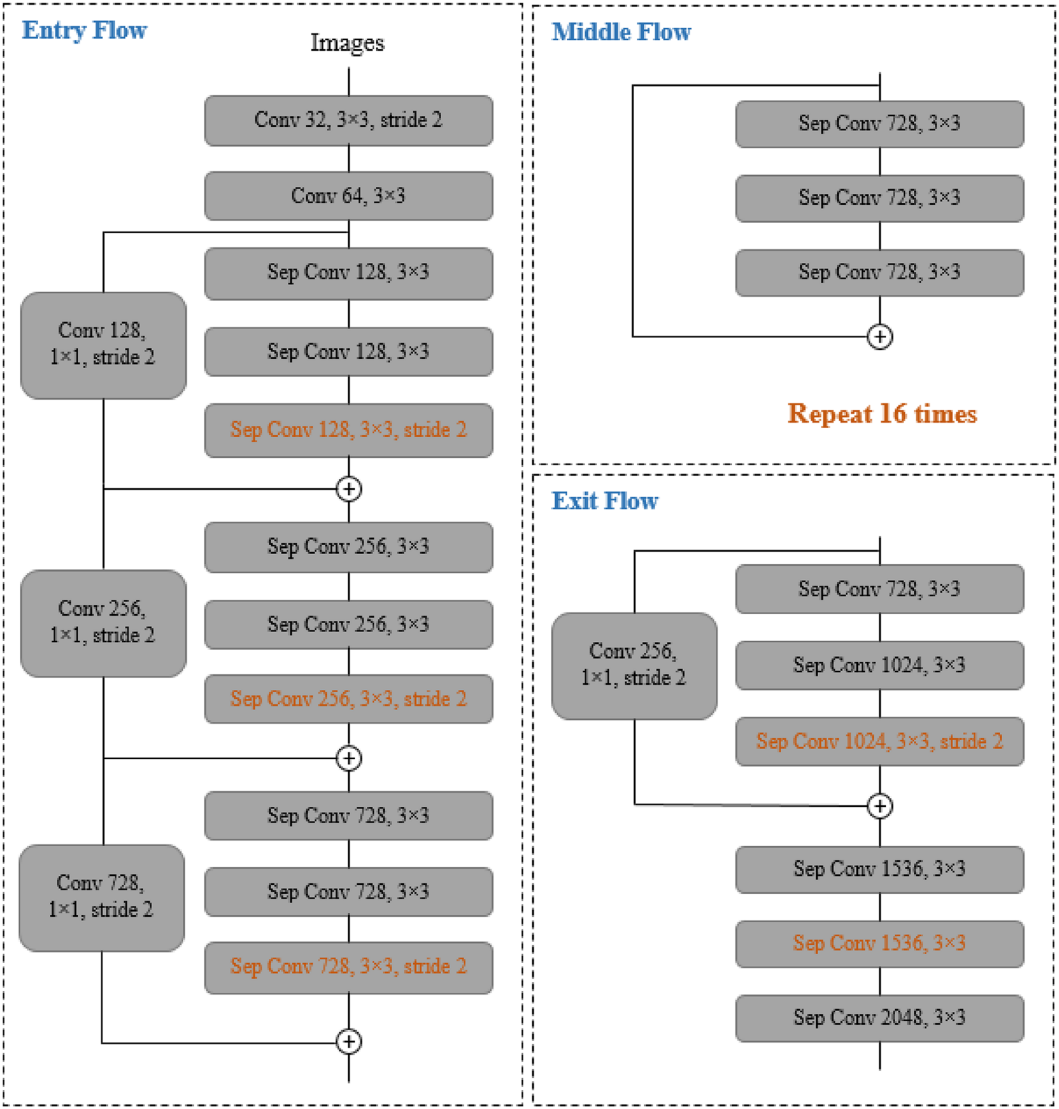
The modified Xception architecture used in DeepLabv3+: (1) has higher number of layers, (2) all max pooling layers are replaced by depthwise seperable convolutions with striding and (3) extra batch normalization and ReLU are added after each 3 × 3 depthwise convolution^42^.

DeepLabv3+ is an extension of the DeepLabv3 model of the DeepLab series. The overall encoder-decoder structure of DeepLabv3+ is represented in Fig. 7. The function of the encoder module is to encode the multi-scale contextual information with help of atrous convolutions, while the decoder module refines the image segmentation results along the object boundaries. The DeepLabv3+ uses a modified Xception model as an encoder as shown in Fig. 8. The earlier DCNN’s had a limitation that the repeated use of max-pooling and striding at every step of these networks, significantly reduced the spatial resolution of the output feature maps^88^. To overcome this problem, atrous convolution, which was originally developed for computing undecimated wavelet transform^89^, is used. It allows the computation of responses at any desired resolution^88^. Convolution operation can be defined with the help of three different parameters—kernel size, stride, and padding while an atrous convolution also known as dilated convolution has an additional parameter called dilation rate (*r*) which is the spacing between the kernel values. If *r* = 1, this special case of atrous convolution becomes equivalent to normal convolution. For a two dimensional signal, for every location *i* on the feature map y and the convolution filter *w*, the atrous convolution is applied over the input feature map x as per the Eq. (12)^42^

12 $$y\left[ i \right] = \mathop \sum \limits_{k} x\left[ {i + r.k} \right]w\left[ k \right]$$

where r = atrous rate, it determines the stride with which the input is sampled.

**Figure 9:**
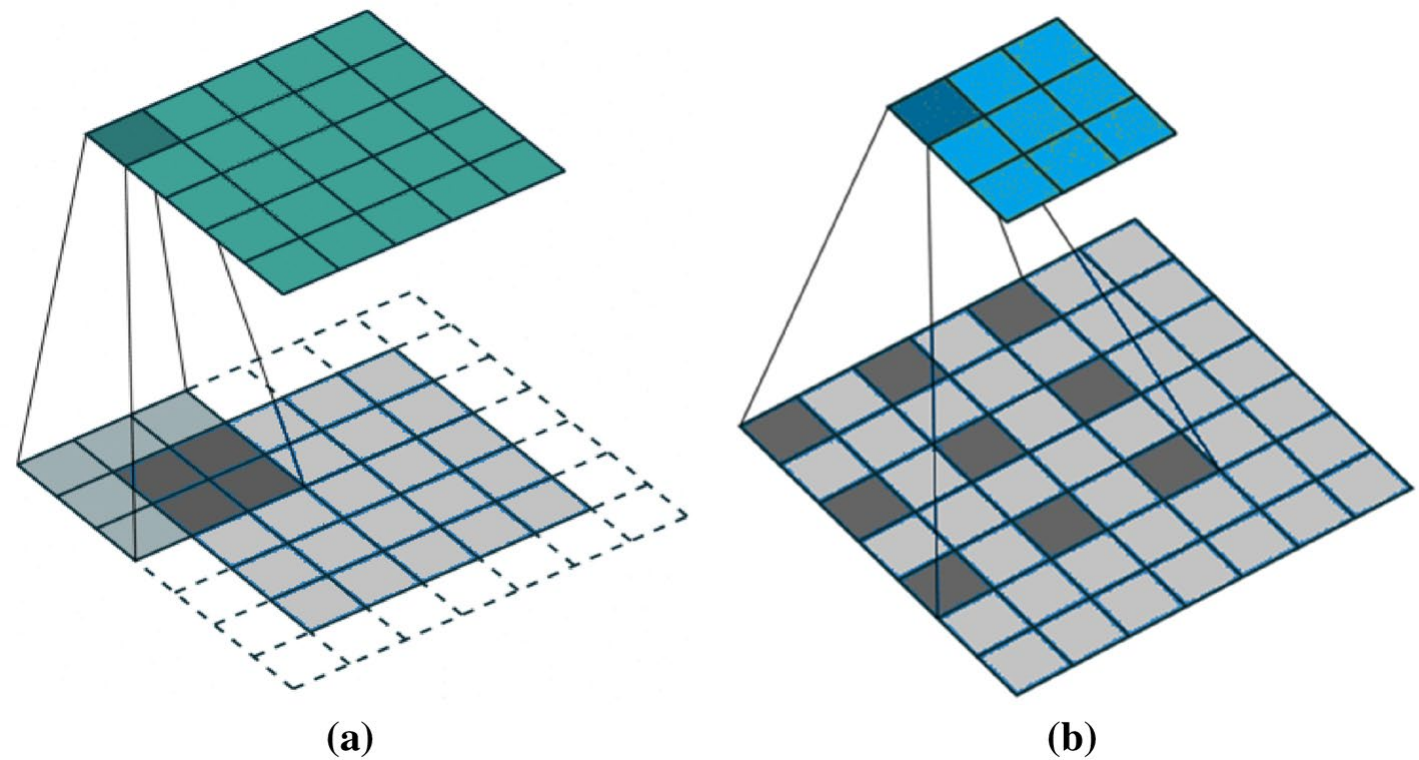
Represents the typical and atrous convolution (**a**) a typical convolution of kernel size = 3, stride = 1 and padding is used to retain the input image size; (**b**) an atrous convolution which has an additional parameter called dilation rate which deletes every alternate row and column.

Atrous convolution allows for a wider field of view at the same computational cost. A comparison between normal convolution and atrous convolution is presented in Fig. 9.

For capturing the contextual information at multiple scales, parallel atrous convolution with different rates which are known as Atrous Spatial Pyramid Pooling (ASPP) was integrated in DeepLabv3. The useful semantic information is present within the last feature map but the information related to object boundaries is missed due to the convolution operation with striding. This problem can be resolved by applying the atrous convolution to extract denser feature maps but this becomes computationally expensive and inefficient. Whereas in the case of encoder-decoder models no features are dilated in the encoder path and they gradually recover the sharp object boundaries in the decoder path leading to faster computations. To combine the advantages of both methods, DeepLabv3+ extends the DeepLabv3 model by adding a simple but effective decoder module for recovering the sharp object boundaries.

It has been reported that by using depthwise separable convolution, the computational complexity in terms of Multiply-Adds is reduced by 33% to 41% while maintaining a similar performance which results in improved speed and accuracy by adapting Xception as a backbone^90,91^.

**Figure 10:**
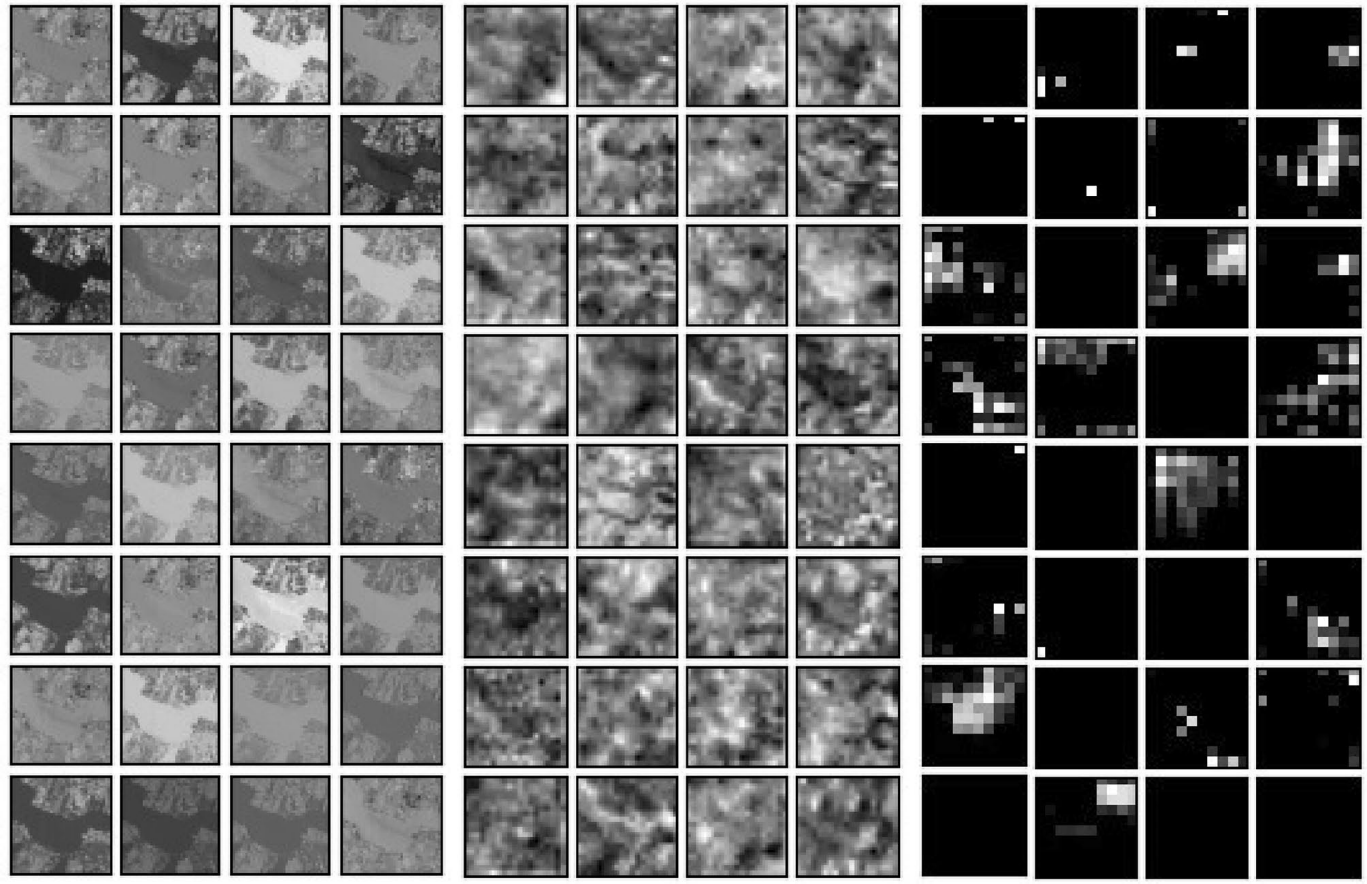
Represents the feature maps extracted from 1st hidden layer of Block1, Block7 and Block14. Feature Maps from all 14 Blocks are presented in Appendix.

The Xception model used as a backbone architecture in the current work has 14 blocks whose details are given in the Appendix. To better understand the working of this deep learning model, a visualization of features maps corresponding to the first hidden layer of each block has been generated. Figure 10 shows the feature maps extracted from Block 1, Block 7, and Block 14 respectively. The features extracted from all 14 blocks are given in the Appendix. It can be noted that the initial layers work on simple and coarser features while the higher layers work on finer details.

This model has implemented using the python programming language, the supporting libraries like numpy, scipy, sklearn, opencv, pandas, and Tensorflow framework that executed using Anaconda (version 4.9.2) on a workstation with NVIDIA Quadro P4000 GPU and 48 GB RAM. It is easy to implement transfer learning using the TensorFlow in Python with a pre-trained model available on the Github page of DeepLabv3+^42^. Since the pre-trained model is trained on the ImageNet dataset which has a much higher number of classes compared to our dataset, few changes have been made in the original DeepLabv3+ code for implementing this model using transfer learning on our custom dataset as discussed in the Appendix.

## Supplementary Information

Supplementary Information.
