# Supplementary material for: Semantic segmentation of PolSAR image data using advanced deep learning model
Source: Sci Rep. 2021 Jul 28;11:15365. doi: 10.1038/s41598-021-94422-y (PMC8319419; doi:10.1038/s41598-021-94422-y)
Supplement: Supplementary file 1 — Supplementary Information. [file 41598_2021_94422_MOESM1_ESM.docx]

**Appendix**

1. **Xception Model Summary**

| Layer (type) | Output Shape | Param # | Connected to |
| --- | --- | --- | --- |
| =========================================================================================== | | | |
| input_1 (InputLayer) | (None, 299, 299, 3) | 0 |  |
| block1_conv1 (Conv2D) | (None, 149, 149, 32) | 864 | input_1[0][0] |
| block1_conv1_bn (BatchNormalization) | (None, 149, 149, 32) | 128 | block1_conv1[0][0] |
| block1_conv1_act (Activation) | (None, 149, 149, 32) | 0 | block1_conv1_bn[0][0] |
| block1_conv2 (Conv2D) | (None, 147, 147, 64) | 18432 | block1_conv1_act[0][0] |
| block1_conv2_bn (BatchNormalization) | (None, 147, 147, 64) | 256 | block1_conv2[0][0] |
| block1_conv2_act (Activation) | (None, 147, 147, 64) | 0 | block1_conv2_bn[0][0] |
| block2_sepconv1 (SeparableConv2) | (None, 147, 147, 128) | 8768 | block1_conv2_act[0][0] |
| block2_sepconv1_bn (BatchNormalization) | (None, 147, 147, 128) | 512 | block2_sepconv1[0][0] |
| block2_sepconv2_act (Activation) | (None, 147, 147, 128) | 0 | block2_sepconv1_bn[0][0] |
| block2_sepconv2 (SeparableConv2) | (None, 147, 147, 128) | 17536 | block2_sepconv2_act[0][0] |
| block2_sepconv2_bn (BatchNormalization) | (None, 147, 147, 128) | 512 | block2_sepconv2[0][0] |
| conv2d (Conv2D) | (None, 74, 74, 128) | 8192 | block1_conv2_act[0][0] |
| block2_pool (MaxPooling2D) | (None, 74, 74, 128) | 0 | block2_sepconv2_bn[0][0] |
| batch_normalization (BatchNormalization) | (None, 74, 74, 128) | 512 | conv2d[0][0] |
| add (Add) | (None, 74, 74, 128) | 0 | block2_pool[0][0] |
|  |  |  | batch_normalization[0][0] |
| block3_sepconv1_act (Activation) | (None, 74, 74, 128) | 0 | add[0][0] |
| block3_sepconv1 (SeparableConv2) | (None, 74, 74, 256) | 33920 | block3_sepconv1_act[0][0] |
| block3_sepconv1_bn (BatchNormalization) | (None, 74, 74, 256) | 1024 | block3_sepconv1[0][0] |
| block3_sepconv2_act (Activation) | (None, 74, 74, 256) | 0 | block3_sepconv1_bn[0][0] |
| block3_sepconv2 (SeparableConv2) | (None, 74, 74, 256) | 67840 | block3_sepconv2_act[0][0] |
| block3_sepconv2_bn (BatchNormalization) | (None, 74, 74, 256) | 1024 | block3_sepconv2[0][0] |
| conv2d_1 (Conv2D) | (None, 37, 37, 256) | 32768 | add[0][0] |
| block3_pool (MaxPooling2D) | (None, 37, 37, 256) | 0 | block3_sepconv2_bn[0][0] |
| batch_normalization_1(BatchNormalization) | (None, 37, 37, 256) | 1024 | conv2d_1[0][0] |
| add_1 (Add) | (None, 37, 37, 256) | 0 | block3_pool[0][0] |
|  |  |  | batch_normalization_1[0][0] |
| block4_sepconv1_act (Activation) | (None, 37, 37, 256) | 0 | add_1[0][0] |
| block4_sepconv1 (SeparableConv2) | (None, 37, 37, 728) | 188672 | block4_sepconv1_act[0][0] |
| block4_sepconv1_bn (BatchNormalization) | (None, 37, 37, 728) | 2912 | block4_sepconv1[0][0] |
| block4_sepconv2_act (Activation) | (None, 37, 37, 728) | 0 | block4_sepconv1_bn[0][0] |
| block4_sepconv2 (SeparableConv2) | (None, 37, 37, 728) | 536536 | block4_sepconv2_act[0][0] |
| block4_sepconv2_bn (BatchNormalization) | (None, 37, 37, 728) | 2912 | block4_sepconv2[0][0] |
| conv2d_2 (Conv2D) | (None, 19, 19, 728) | 186368 | add_1[0][0] |
| block4_pool (MaxPooling2D) | (None, 19, 19, 728) | 0 | block4_sepconv2_bn[0][0] |
| batch_normalization_2(BatchNormalization) | (None, 19, 19, 728) | 2912 | conv2d_2[0][0] |
| add_2 (Add) | (None, 19, 19, 728) | 0 | block4_pool[0][0] |
|  |  |  | batch_normalization_2[0][0] |
| block5_sepconv1_act (Activation) | (None, 19, 19, 728) | 0 | add_2[0][0] |
| block5_sepconv1 (SeparableConv2) | (None, 19, 19, 728) | 536536 | block5_sepconv1_act[0][0] |
| block5_sepconv1_bn (BatchNormalization) | (None, 19, 19, 728) | 2912 | block5_sepconv1[0][0] |
| block5_sepconv2_act (Activation) | (None, 19, 19, 728) | 0 | block5_sepconv1_bn[0][0] |
| block5_sepconv2 (SeparableConv2) | (None, 19, 19, 728) | 536536 | block5_sepconv2_act[0][0] |
| block5_sepconv2_bn (BatchNormalization) | (None, 19, 19, 728) | 2912 | block5_sepconv2[0][0] |
| block5_sepconv3_act (Activation) | (None, 19, 19, 728) | 0 | block5_sepconv2_bn[0][0] |
| block5_sepconv3 (SeparableConv2) | (None, 19, 19, 728) | 536536 | block5_sepconv3_act[0][0] |
| block5_sepconv3_bn (BatchNormalization) | (None, 19, 19, 728) | 2912 | block5_sepconv3[0][0] |
| add_3 (Add) | (None, 19, 19, 728) | 0 | block5_sepconv3_bn[0][0] |
|  |  |  | add_2[0][0] |
| block6_sepconv1_act (Activation) | (None, 19, 19, 728) | 0 | add_3[0][0] |
| block6_sepconv1 (SeparableConv2) | (None, 19, 19, 728) | 536536 | block6_sepconv1_act[0][0] |
| block6_sepconv1_bn (BatchNormalization) | (None, 19, 19, 728) | 2912 | block6_sepconv1[0][0] |
| block6_sepconv2_act (Activation) | (None, 19, 19, 728) | 0 | block6_sepconv1_bn[0][0] |
| block6_sepconv2 (SeparableConv2) | (None, 19, 19, 728) | 536536 | block6_sepconv2_act[0][0] |
| block6_sepconv2_bn (BatchNormalization) | (None, 19, 19, 728) | 2912 | block6_sepconv2[0][0] |
| block6_sepconv3_act (Activation) | (None, 19, 19, 728) | 0 | block6_sepconv2_bn[0][0] |
| block6_sepconv3 (SeparableConv2) | (None, 19, 19, 728) | 536536 | block6_sepconv3_act[0][0] |
| block6_sepconv3_bn (BatchNormalization) | (None, 19, 19, 728) | 2912 | block6_sepconv3[0][0] |
| add_4 (Add) | (None, 19, 19, 728) | 0 | block6_sepconv3_bn[0][0] |
|  |  |  | add_3[0][0] |
| block7_sepconv1_act (Activation) | (None, 19, 19, 728) | 0 | add_4[0][0] |
| block7_sepconv1 (SeparableConv2) | (None, 19, 19, 728) | 536536 | block7_sepconv1_act[0][0] |
| block7_sepconv1_bn (BatchNormalization) | (None, 19, 19, 728) | 2912 | block7_sepconv1[0][0] |
| block7_sepconv2_act (Activation) | (None, 19, 19, 728) | 0 | block7_sepconv1_bn[0][0] |
| block7_sepconv2 (SeparableConv2) | (None, 19, 19, 728) | 536536 | block7_sepconv2_act[0][0] |
| block7_sepconv2_bn (BatchNormalization) | (None, 19, 19, 728) | 2912 | block7_sepconv2[0][0] |
| block7_sepconv3_act (Activation) | (None, 19, 19, 728) | 0 | block7_sepconv2_bn[0][0] |
| block7_sepconv3 (SeparableConv2) | (None, 19, 19, 728) | 536536 | block7_sepconv3_act[0][0] |
| block7_sepconv3_bn (BatchNormalization) | (None, 19, 19, 728) | 2912 | block7_sepconv3[0][0] |
| add_5 (Add) | (None, 19, 19, 728) | 0 | block7_sepconv3_bn[0][0] |
|  |  |  | add_4[0][0] |
| block8_sepconv1_act (Activation) | (None, 19, 19, 728) | 0 | add_5[0][0] |
| block8_sepconv1 (SeparableConv2) | (None, 19, 19, 728) | 536536 | block8_sepconv1_act[0][0] |
| block8_sepconv1_bn (BatchNormalization) | (None, 19, 19, 728) | 2912 | block8_sepconv1[0][0] |
| block8_sepconv2_act (Activation) | (None, 19, 19, 728) | 0 | block8_sepconv1_bn[0][0] |
| block8_sepconv2 (SeparableConv2) | (None, 19, 19, 728) | 536536 | block8_sepconv2_act[0][0] |
| block8_sepconv2_bn (BatchNormalization) | (None, 19, 19, 728) | 2912 | block8_sepconv2[0][0] |
| block8_sepconv3_act (Activation) | (None, 19, 19, 728) | 0 | block8_sepconv2_bn[0][0] |
| block8_sepconv3 (SeparableConv2) | (None, 19, 19, 728) | 536536 | block8_sepconv3_act[0][0] |
| block8_sepconv3_bn (BatchNormalization) | (None, 19, 19, 728) | 2912 | block8_sepconv3[0][0] |
| add_6 (Add) | (None, 19, 19, 728) | 0 | block8_sepconv3_bn[0][0] |
|  |  |  | add_5[0][0] |
| block9_sepconv1_act (Activation) | (None, 19, 19, 728) | 0 | add_6[0][0] |
| block9_sepconv1 (SeparableConv2) | (None, 19, 19, 728) | 536536 | block9_sepconv1_act[0][0] |
| block9_sepconv1_bn (BatchNormalization) | (None, 19, 19, 728) | 2912 | block9_sepconv1[0][0] |
| block9_sepconv2_act (Activation) | (None, 19, 19, 728) | 0 | block9_sepconv1_bn[0][0] |
| block9_sepconv2 (SeparableConv2) | (None, 19, 19, 728) | 536536 | block9_sepconv2_act[0][0] |
| block9_sepconv2_bn (BatchNormalization) | (None, 19, 19, 728) | 2912 | block9_sepconv2[0][0] |
| block9_sepconv3_act (Activation) | (None, 19, 19, 728) | 0 | block9_sepconv2_bn[0][0] |
| block9_sepconv3 (SeparableConv2) | (None, 19, 19, 728) | 536536 | block9_sepconv3_act[0][0] |
| block9_sepconv3_bn (BatchNormalization) | (None, 19, 19, 728) | 2912 | block9_sepconv3[0][0] |
| add_7 (Add) | (None, 19, 19, 728) | 0 | block9_sepconv3_bn[0][0] |
|  |  |  | add_6[0][0] |
| block10_sepconv1_act (Activation) | (None, 19, 19, 728) | 0 | add_7[0][0] |
| block10_sepconv1 (SeparableConv2) | (None, 19, 19, 728) | 536536 | block10_sepconv1_act[0][0] |
| block10_sepconv1_bn (BatchNormalization) | (None, 19, 19, 728) | 2912 | block10_sepconv1[0][0] |
| block10_sepconv2_act (Activation) | (None, 19, 19, 728) | 0 | block10_sepconv1_bn[0][0] |
| block10_sepconv2 (SeparableConv2) | (None, 19, 19, 728) | 536536 | block10_sepconv2_act[0][0] |
| block10_sepconv2_bn (BatchNormalization) | (None, 19, 19, 728) | 2912 | block10_sepconv2[0][0] |
| block10_sepconv3_act (Activation) | (None, 19, 19, 728) | 0 | block10_sepconv2_bn[0][0] |
| block10_sepconv3 (SeparableConv2) | (None, 19, 19, 728) | 536536 | block10_sepconv3_act[0][0] |
| block10_sepconv3_bn (BatchNormalization) | (None, 19, 19, 728) | 2912 | block10_sepconv3[0][0] |
| add_8 (Add) | (None, 19, 19, 728) | 0 | block10_sepconv3_bn[0][0] |
|  |  |  | add_7[0][0] |
| block11_sepconv1_act (Activation) | (None, 19, 19, 728) | 0 | add_8[0][0] |
| block11_sepconv1 (SeparableConv2) | (None, 19, 19, 728) | 536536 | block11_sepconv1_act[0][0] |
| block11_sepconv1_bn (BatchNormalization) | (None, 19, 19, 728) | 2912 | block11_sepconv1[0][0] |
| block11_sepconv2_act (Activation) | (None, 19, 19, 728) | 0 | block11_sepconv1_bn[0][0] |
| block11_sepconv2 (SeparableConv2) | (None, 19, 19, 728) | 536536 | block11_sepconv2_act[0][0] |
| block11_sepconv2_bn (BatchNormalization) | (None, 19, 19, 728) | 2912 | block11_sepconv2[0][0] |
| block11_sepconv3_act (Activation) | (None, 19, 19, 728) | 0 | block11_sepconv2_bn[0][0] |
| block11_sepconv3 (SeparableConv2) | (None, 19, 19, 728) | 536536 | block11_sepconv3_act[0][0] |
| block11_sepconv3_bn (BatchNormalization) | (None, 19, 19, 728) | 2912 | block11_sepconv3[0][0] |
| add_9 (Add) | (None, 19, 19, 728) | 0 | block11_sepconv3_bn[0][0] |
|  |  |  | add_8[0][0] |
| block12_sepconv1_act (Activation) | (None, 19, 19, 728) | 0 | add_9[0][0] |
| block12_sepconv1 (SeparableConv2) | (None, 19, 19, 728) | 536536 | block12_sepconv1_act[0][0] |
| block12_sepconv1_bn (BatchNormalization) | (None, 19, 19, 728) | 2912 | block12_sepconv1[0][0] |
| block12_sepconv2_act (Activation) | (None, 19, 19, 728) | 0 | block12_sepconv1_bn[0][0] |
| block12_sepconv2 (SeparableConv2) | (None, 19, 19, 728) | 536536 | block12_sepconv2_act[0][0] |
| block12_sepconv2_bn (BatchNormalization) | (None, 19, 19, 728) | 2912 | block12_sepconv2[0][0] |
| block12_sepconv3_act (Activation) | (None, 19, 19, 728) | 0 | block12_sepconv2_bn[0][0] |
| block12_sepconv3 (SeparableConv2) | (None, 19, 19, 728) | 536536 | block12_sepconv3_act[0][0] |
| block12_sepconv3_bn (BatchNormalization) | (None, 19, 19, 728) | 2912 | block12_sepconv3[0][0] |
| add_10 (Add) | (None, 19, 19, 728) | 0 | block12_sepconv3_bn[0][0] |
|  |  |  | add_9[0][0] |
| block13_sepconv1_act (Activation) | (None, 19, 19, 728) | 0 | add_10[0][0] |
| block13_sepconv1 (SeparableConv2) | (None, 19, 19, 728) | 536536 | block13_sepconv1_act[0][0] |
| block13_sepconv1_bn (BatchNormalization) | (None, 19, 19, 728) | 2912 | block13_sepconv1[0][0] |
| block13_sepconv2_act (Activation) | (None, 19, 19, 728) | 0 | block13_sepconv1_bn[0][0] |
| block13_sepconv2 (SeparableConv2) | (None, 19, 19, 1024) | 752024 | block13_sepconv2_act[0][0] |
| block13_sepconv2_bn (BatchNormalization) | (None, 19, 19, 1024) | 4096 | block13_sepconv2[0][0] |
| conv2d_3 (Conv2D) | (None, 10, 10, 1024) | 745472 | add_10[0][0] |
| block13_pool (MaxPooling2D) | (None, 10, 10, 1024) | 0 | block13_sepconv2_bn[0][0] |
| batch_normalization_3 (BatchNormalization) | (None, 10, 10, 1024) | 4096 | conv2d_3[0][0] |
| add_11 (Add) | (None, 10, 10, 1024) | 0 | block13_pool[0][0] |
|  |  |  | batch_normalization_3[0][0] |
| block14_sepconv1 (SeparableConv2) | (None, 10, 10, 1536) | 1582080 | add_11[0][0] |
| block14_sepconv1_bn (BatchNormalization) | (None, 10, 10, 1536) | 6144 | block14_sepconv1[0][0] |
| block14_sepconv1_act (Activation) | (None, 10, 10, 1536) | 0 | block14_sepconv1_bn[0][0] |
| block14_sepconv2 (SeparableConv2) | (None, 10, 10, 2048) | 3159552 | block14_sepconv1_act[0][0] |
| block14_sepconv2_bn (BatchNormalization) | (None, 10, 10, 2048) | 8192 | block14_sepconv2[0][0] |
| block14_sepconv2_act (Activation) | (None, 10, 10, 2048) | 0 | block14_sepconv2_bn[0][0] |
| avg_pool (GlobalAveragePooling2D) | (None, 2048) | 0 | block14_sepconv2_act[0][0] |
| predictions (Dense) | (None, 1000) | 2049000 | avg_pool[0][0] |
| ======================================================================================== | | | |
| Total params: 22,910,480 |  |  |  |
| Trainable params: 22,855,952 |  |  |  |
| Non-trainable params: 54,528 |  |  |  |

1. **Training Custom Dataset using Transfer Learning**

For training the DeepLab model on a custom dataset, it is required to convert the data to TFRecord format. TFRecord is a simple binary file format for storing data which helps in efficient computing because it takes lesser disk space and can be read faster.

Firstly, we need to store our data as per the below-mentioned directory structure:


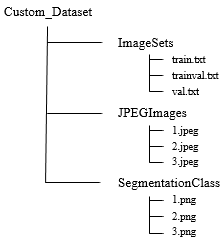


The train.txt, val.txt, and trainval.txt contains the names of image patches to be used for training, validation, and all images combined (training+validation) respectively. The JPEGImages folder should contain the false-color composite image patches for training and validation. The SegmentationClass folder should contain the annotated labels.

Once, the directory is set, it is required to run the build_voc2012_data.py with updated flag values as per the directory. This converts the data to TFRecord format and it is saved to the location pointed by ‘- output_dir’. The segmentation_dataset.py requires updation (addition of _SAR_INFORMATION) as shown below:


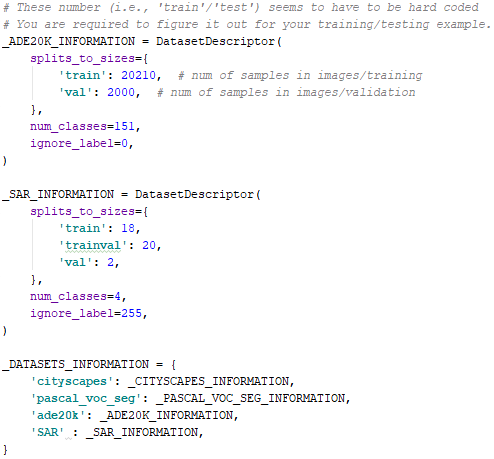


Additional Code

Once this is done, we can run train.py, and vis.py codes using the commands given on the GitHub page of DeepLabv3+ for training, evaluation, and visualization of results. Apart from this one more modification has been done in the visualization part by adding a custom colormap for better inference of the segmentation results. The python code for the same can be found below:

**from** __future__ **import** absolute_import
**from** __future__ **import** division
**from** __future__ **import** print_function
**import** numpy **as** np
**from** six.moves **import** range

*# Dataset name.*_CUSTOM = **'SAR'***# Max number of entries in the colormap for each dataset.*_DATASET_MAX_ENTRIES = {
 _CUSTOM: 4,
}

**def** create_custom_label_colormap():
 colormap = np.zeros((5, 3), dtype=np.uint8)
 colormap[0] = [120, 255, 120] *#GROUND* colormap[1] = [0, 100, 0] *#FOREST* colormap[2] = [255, 175, 175] *#URBAN* colormap[3] = [0, 0, 255] *#WATER* **return** colormap

**def** get_custom_name():
 **return** _CUSTOM

**def** create_label_colormap(dataset=_CUSTOM):
 **if** dataset == _CUSTOM:
 **return** create_custom_label_colormap()
 **else**:
 **raise** ValueError(**'Unsupported dataset.'**)

**def** label_to_color_image(label, dataset=_CUSTOM):
 **if** label.ndim != 2:
 **raise** ValueError(**'Expect 2-D input label. Got {}'**.format(label.shape))

**if** np.max(label) >= _DATASET_MAX_ENTRIES[dataset]:
 **raise** ValueError(**'label value too large: {} >= {}.'**.format(np.max(label),

_DATASET_MAX_ENTRIES[dataset]))
 colormap = create_label_colormap(dataset)
 **return** colormap[label]

**def** get_dataset_colormap_max_entries(dataset):
 **return** _DATASET_MAX_ENTRIES[dataset]

1. **Feature Visualization**


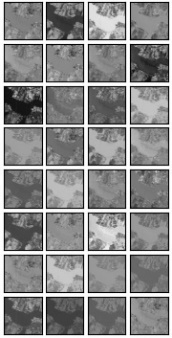

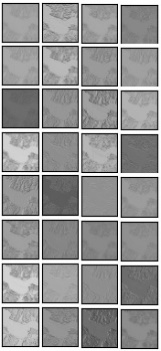

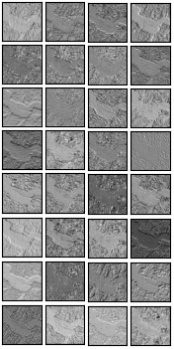

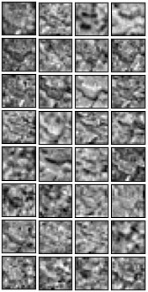

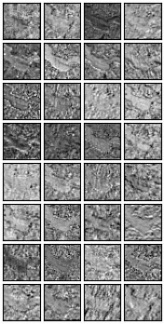


Block 1

Block 2

Block 3

Block 4

Block 5


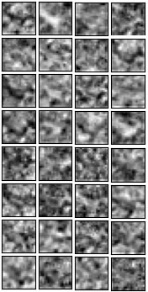

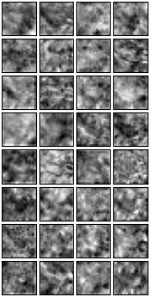


Block 6

Block 7


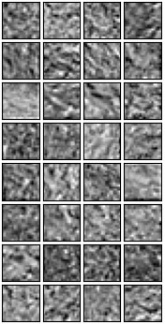

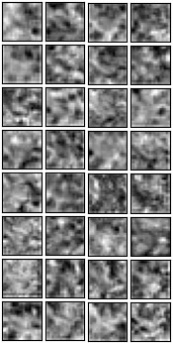

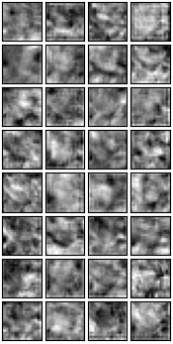


Block 8

Block 9

Block 10

Visualization of the Feature Maps extracted block-wise using the Xception Model corresponding to the 1^st^ hidden layer of every block for test patch 1.


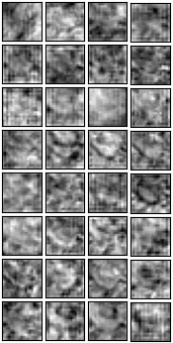

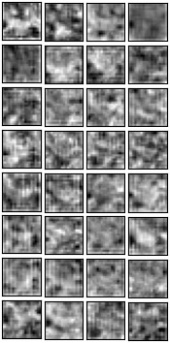

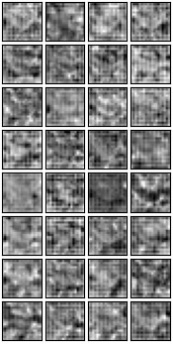

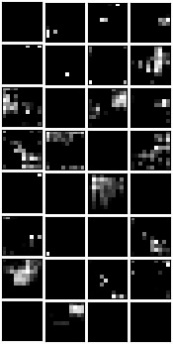


Block 11

Block 12

Block 13

Block 14
